# 

**Published:** 1908-08-15

**Authors:** 


### BOOKS RECEIVED.

Baillière, Tindall and Cox.

"The National Physique." By A. Stayt Dutton, L.R.C.P.

Cassell and Co., Ltd.

"The Renal Function in Urinary Surgery." By Thomson Walker, M.B.

The Sanitary Publishing Co., Ltd.

"Annotated By-laws as to House Drainage, etc." By Gerard J. G. Jensen, C.E.

The Scientific Press, Ltd.

"The Care and Nursing of the Insane." By Percy J. Baily, M.B.

"Science and Empiricism." By H. C. Daniel.

In the annual report of the medical officer of health for the Borough of Finsbury appears an account of the successful use of modified dried milk—"Glaxo"—in a number of children (fifty-three in all) who could not obtain the depot milk and who could not be fed on breast milk. This substance is the dried essence of whole milk, suitably modified, and when re-constituted forms a liquid milk, having much the same constituent parts as ordinary modified milk. Being in powder form it can be kept for long periods, and is not affected by hot weather. The report shows that this preparation is an excellent substitute for much of the milk upon which infants are now fed; the progress of the children under observation, as attested by gain in body-weight, being very satisfactory.

